# Pruritus related to trastuzumab and pertuzumab in HER2 + breast cancer patients

**DOI:** 10.1007/s10549-023-07143-3

**Published:** 2023-10-13

**Authors:** Stephanie Gu, Stephen Dusza, Elizabeth Quigley, Helen Haliasos, Alina Markova, Michael Marchetti, Andrea P. Moy, Chau Dang, Shanu Modi, Diana Lake, Sarah Noor, Mario E. Lacouture

**Affiliations:** 1 Dermatology Service, Department of Medicine, Memorial Sloan Kettering Cancer Center, 545 East 73rd Street, New York, NY 10021 USA; 2 Dermatopathology Service, Department of Pathology and Laboratory Medicine, Memorial Sloan Kettering Cancer Center, New York, USA; 3 Breast Service, Department of Medicine, Memorial Sloan Kettering Cancer Center, New York, USA; 4 Department of Dermatology, Weill Cornell Medical College, New York, USA

**Keywords:** Pruritus, Dermatologic adverse events, Trastuzumab, Pertuzumab, Rash

## Abstract

**Purpose:**

The combination of trastuzumab and pertuzumab (HP) as part of a taxane-based regimen has shown benefit in the adjuvant and metastatic HER2 + breast cancer setting. In the CLEOPATRA trial, pruritus was reported in 11-17.6% of patients. The clinical phenotype and potential treatment strategies for this event have not been reported.

**Methods:**

A retrospective review of 2583 patients receiving trastuzumab and pertuzumab for the treatment of HER2 + breast cancer from 11/23/2011 to 6/21/2021 was performed at Memorial Sloan Kettering Cancer Center (MSKCC). Patient demographics, pruritus characteristics, and treatments as documented in the electronic medical record (EMR) were included in this analysis.

**Results:**

Of 2583 pts treated with HP, 122 (4.72%) with pruritus were identified. On average, patients experienced pruritus 319.0 days (8-3171) after initiation of HP. The upper extremities (67.4%), back (29.3%), lower extremities (17.4%), and shoulders (14.1%) were the most commonly affected regions. Grade 1/2 pruritus (97.6%) occurred in most cases. Patients responded primarily to treatment with topical steroids (52.2%), antihistamines (29.9%), emollients (20.9%), and gabapentinoids (16.4%). Of those with pruritus, 4 patients (3.3%) required treatment interruption or discontinuation.

**Conclusions:**

Pruritus is uncommon in patients on trastuzumab and pertuzumab, generally a chronic condition, with gabapentinoids or antihistamines representing effective therapies.

## Introduction

Human epidermal growth factor 2 (HER2) is a transmembrane receptor that plays an integral role in the control of epithelial cell growth and differentiation [1]. Amplification of this receptor has been reported in many forms of cancer and is generally associated with poor prognosis and increased disease recurrence. Trastuzumab and pertuzumab are two FDA approved humanized anti-HER2 antibodies that are designed to target this receptor. These medications work synergistically by binding to and inhibiting the HER2 receptor, each at different sites, ultimately inducing antibody dependent cell mediated cytotoxicity and tumor death.

The addition of pertuzumab to trastuzumab in a taxane-based regimen demonstrated even greater therapeutic efficacy and is now being widely used for the treatment of HER2-positive breast cancer [2–4]. In addition, this regimen is FDA-approved for use in combination with docetaxel for HER2-positive metastatic breast cancer, or in combination with chemotherapy for adjuvant and neoadjuvant therapies for HER2-positive locally advanced, inflammatory, or early-stage breast cancer [5–7]. To date, there is limited discussion of skin toxicities in the literature, despite up to 20–30% of patients reporting rash and pruritus in clinical trials [4, 6, 8]. A previous systematic review showed that treatment with pertuzumab-based therapy significantly increased risk of rash development, with all grade rash occurring in 24.6% of patients [9]. Skin and nail infections related to HP plus docetaxel combination therapy have also been previously described [10]. In our own clinical experience, many patients on these therapies present to dermatology with pruritus, often without concurrent rash. To date, there is no discussion in the literature on this toxicity – therefore, in this single-center study, we seek to characterize the pruritus these patients experience and discuss the clinical presentation and treatment of this bothersome symptom.

## Methods

**Fig. 1:**
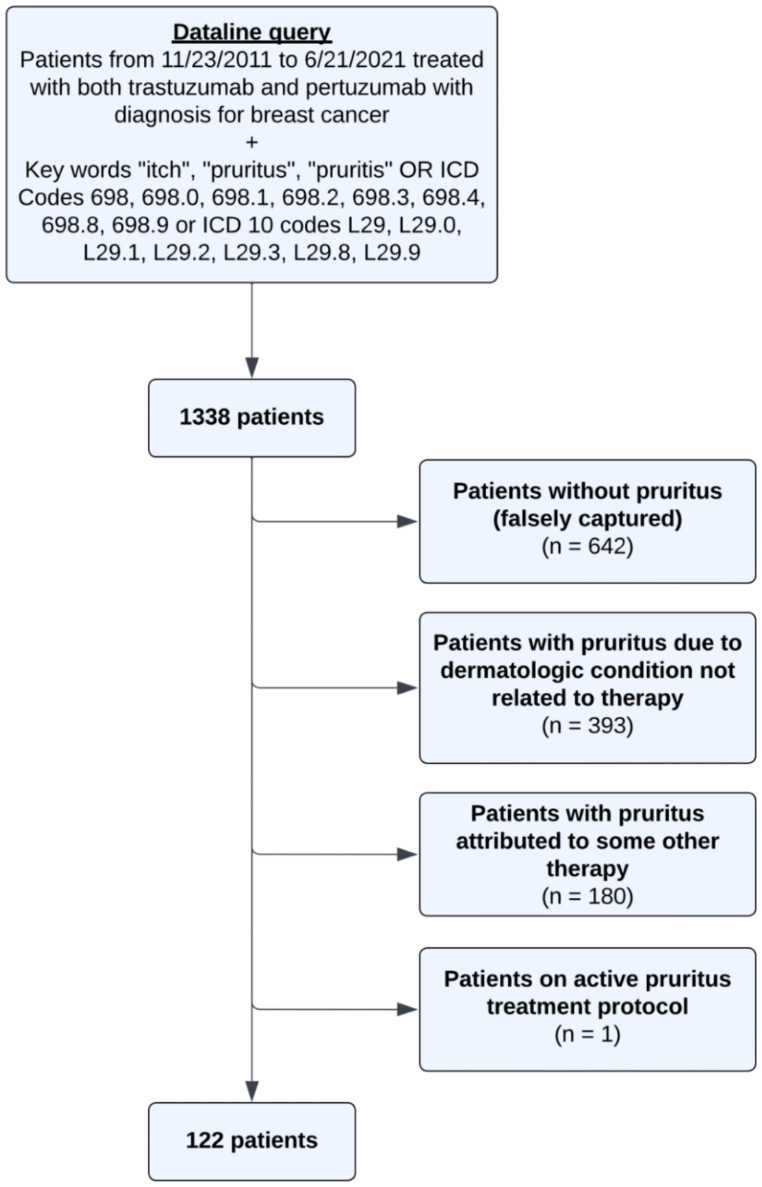
Study schema

This study was conducted under a MSK institutional review board-approved protocol #16–458. A retrospective chart review of patients receiving both trastuzumab and pertuzumab for the treatment of HER2 positive breast cancer (SEER category 1) from 11/23/2011 to 6/21/2021 was performed at Memorial Sloan Kettering Cancer Center (MSKCC). In total, 2583 patients were on both trastuzumab and pertuzumab during this time. Patients were then narrowed down using a database query, which identified electronic medical records containing the key words “itch”, “pruritus”, and/or “pruritis” within breast or dermatology clinical visit notes and/or patients with itch associated ICD 9/10 codes (n = 1338). Electronic records were then reviewed for documentation of itch or pruritus. Patients who did not have pruritus (n = 842), had pruritus due to other treatments (n = 80) or dermatologic conditions not related to therapy (n = 293), and were on an active pruritus treatment protocol (n = 1) were excluded from the analysis (Fig. 1).

Complete blood count, metabolic panels and inflammatory marker data was collected +/- 14 days within onset of pruritus. Only labs relevant to the development of pruritus were included in our results. The onset date of pruritus was determined based either on the first date of documentation or estimated based on the patient history described in the clinical record. Grading of pruritus and rash were based on Common Terminology Criteria for Adverse Events (CTCAE v5.0 for onset after 11/27/2017; corresponding v4.0 for prior onsets). Histopathology of any skin biopsies was examined by a dermatopathologist.

Descriptive statistics were used to describe patient demographics and pruritus characteristics. A figure describing anatomic distribution of pruritus was generated using RStudio.

## Results

### Demographics

A total of 122 female patients (median age at pruritus onset 54.5, range 28–88) with a diagnosis of HER2 + breast cancer who experienced pruritus attributed to HP from 11/23/2011 to 6/21/2021 were included in this study (Table 1). The reported incidence of pruritus at our institution was 4.72%. Most patients were white (68.0%), Asian (12.3%) and black/African American (10.7%). Patients with breast cancer stages I-IV were included. Patients with stage II and stage IV disease comprised the largest groups (38.5% each). Stage I and Stage III disease were less common at 13.9% and 9.0%, respectively. Most patients’ primary regimen was doxorubicin and cyclophosphamide followed by paclitaxel, trastuzumab and pertuzumab (AC THP) (44.3%) or paclitaxel, trastuzumab and pertuzumab (THP) (41.0%). Less common regimens included docetaxel, carboplatin, trastuzumab, and pertuzumab (TCHP) (7.4%) and HP only (2.5%). Vinorelbine was used infrequently as a substitute in patients who did not tolerate initial trials with paclitaxel. Gemcitabine, trastuzumab, and pertuzumab (GHP) and doxorubicin, cyclophosphamide, methotrexate, fluorouracil, paclitaxel, trastuzumab, and pertuzumab (AC CMF THP) regimens were used in one patient (0.8%) each.

**Table 1:** Characteristics of study population (n = 122)

		n (%)
Sex	Male	0 (0)
	Female	122 (100.0)
Race	White	83 (68.0)
	Asian	15 (12.3)
	Black or African American	13 (10.7)
	Other/Unknown	11 (9.0)
Ethnicity	Non-Hispanic	108 (88.5)
	Hispanic	9 (7.4)
	Unknown	5 (4.1)
Stage of disease	Stage I	17 (13.9)
	Stage II	47 (38.5)
	Stage III	11 (9.0)
	Stage IV	47 (38.5)
Breast cancer regimen	AC THP	54 (44.3)
	THP	50 (41.0)
	TCHP	9 (7.4)
	VHP	4 (3.3)
	HP	3 (2.5)
	GHP	1 (0.8)
	AC CMF THP	1 (0.8)
Breast cancer regimen at time of pruritus onset	HP	68 (55.7)
	THP	47 (38.5)
	TCHP	4 (3.3)
	VHP	1 (0.8)
	GHP	1 (0.8)
	CMF THP	1 (0.8)
Concurrent cancer treatment	Cytotoxic	49 (40.2)
	Hormonal	25 (20.5)
	Hormonal + cytotoxic	2 (1.6)
	None	46 (37.7)

Table key:

ACTHP: doxorubicin, cyclophosphamide → paclitaxel, trastuzumab, pertuzumab.

THP: paclitaxel, trastuzumab, pertuzumab.

TCHP: docetaxel, carboplatin, trastuzumab, and pertuzumab.

VHP: vinorelbine, trastuzumab, pertuzumab.

GHP: gemcitabine, trastuzumab, pertuzumab.

AC CMF THP: doxorubicin, cyclophosphamide → cyclophosphamide, methotrexate, fluorauracil → paclitaxel, trastuzumab, pertuzumab.

At the time of pruritus development, most patients had completed their cytotoxic chemotherapies and were on HP therapy (55.7%). The remaining patients were still receiving concurrent paclitaxel (38.5%), docetaxel/carboplatin (3.3%), vinorelbine (0.8%), gemcitabine (0.8%), and cyclophosphamide/methotrexate/fluorouracil (0.8%). Twenty-seven patients (22.1%) were also on tamoxifen or aromatase inhibitors for the treatment of their breast cancer at the time of pruritus onset.

### Cutaneous toxicity

**Fig. 2:**
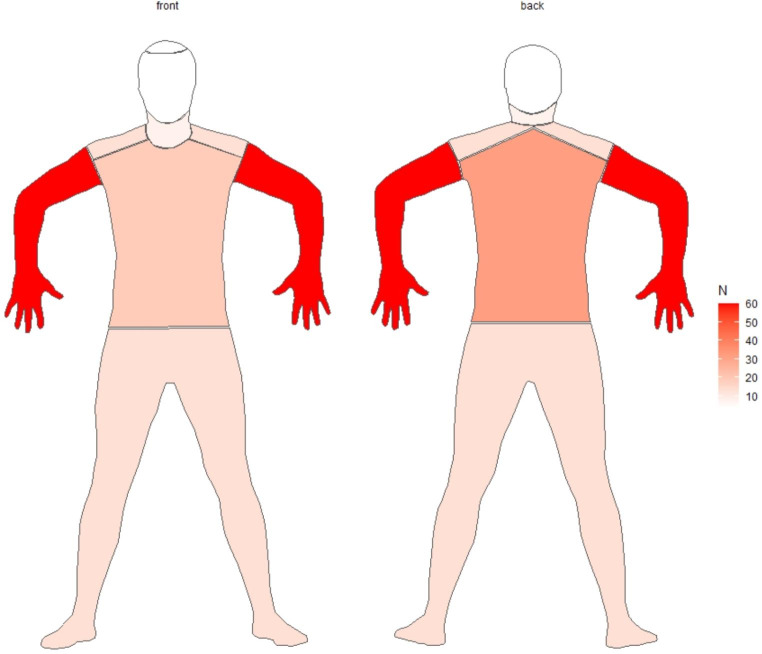
Anatomic distribution of pruritus

On average, patients experienced pruritus 319.0 days (8-3171) after initiation of HP combination therapy. The anatomic distribution of symptoms was described in 92 (75.4%) of our patients (Fig. 2). Among them, the most affected areas were the upper extremities (67.4%), back (29.3%), lower extremities (17.4%), shoulders (14.1%), chest (14.1%), and neck (8.7%). Less common sites of involvement included the torso (7.6%), face (6.5%), scalp (4.3%), and axillae (10.9%). Forty-two patients (34.4%) had documentation of pruritus grade. Among them, grade 2 (61.9%) was the most common followed by grade 1 (35.7%) and grade 3 (2.4%). Eighteen patients (14.8%) experienced a concurrent rash. These were maculopapular (50%), eczematous (22.2%), and acneiform (27.8%) in morphology (Table 2).

**Table 2:** Characteristics of pruritus (n = 122)

		n (%)
Grade	G1	15 (12.3)
	G2	26 (21.3)
	G3	1 (0.8)
	Not documented	80 (65.6)
Concurrent rash	Maculopapular	9 (7.4)
	Acneiform	5 (4.1)
	Eczematous	4 (3.3)
	None	104 (85.2)
Anatomic distribution	Upper extremities	62 (50.8)
	Back	27 (22.1)
	Lower extremities	16 (13.1)
	Shoulders	13 (10.7)
	Chest	13 (10.7)
	Neck	8 (6.6)
	Torso	7 (5.7)
	Face	6 (4.9)
	Scalp	4 (3.3)
	Axillae	1 (0.8)
	Not documented	30 (24.6)
Response to treatment by oncology and/or dermatology	Improved	67 (54.9)
	No response	7 (5.7)
	Not documented	48 (39.3)
Required interruption of HP	No	118 (96.7)
	Yes	4 (3.3)

Table key:

G1: CTCAE Grade 1

G2: CTCAE Grade 2

G3: CTCAE Grade 3

### Laboratory values

Complete blood counts (CBC) were obtained from 105 patients (86.1%). Hemoglobin was under the normal level in 42 (40%) of patients. Platelet counts were above the normal range in 9 patients (8.6%). One hundred and two (83.6%) had absolute eosinophil levels measured as part of their CBC. One of these patients had an elevated level of 1.2.

Metabolic panels were performed in 84 (68.9%) patients. Glomerular filtration rate was decreased below the normal range ( > = 60 mL/min/1.73m^2^) in 5 (6.0%) patients and ranged from 25 to 52 mL/min/1.73^2^. Alkaline phosphatase was measured in 84 (68.9%) patients; 5 patients (6.0%) had levels above the normal range. Aspartate transaminase (AST) and alanine transaminase (ALT) levels were measured in 80 patients (65.6%). AST was elevated above normal levels in 8 patients (9.5%) and ALT was elevated above normal in 2 patients (2.4%).

Of those patients with circulating cytokine levels, four patients (3.3%) had interleukin 5 (IL5) levels measured and all except one patient, who had a level of 4.5 pg/mL, were within the normal range. Fifteen patients (12.3%) had their Immunoglobulin E (IgE) levels measured. Only 3 patients (20.0%) had elevations (Table 3).

**Table 3:** Laboratory values of patient cohort

n		
105	Hemoglobin, g/dL, mean (range)	11.6 (7.7–16.5)
105	Platelets, K/mcL, mean (range)	273.5 (99–535)
102	Eosinophils (absolute), K/mcL, mean (range)	0.14 (0-1.2)
84	Alkaline phosphatase, U/L, mean (range)	82.8 (33–190)
80	Aspartate transaminase, U/L, mean (range)	27.8 (13–295)
80	Alanine transaminase, U/L, mean (range)	35.6 (10–644)
4	Interleukin-5, pg/mL, median (range)	< 1 (< 1-4.5)
15	Immunoglobulin E, kU/L, mean (range)	86 (2-352)

Normal ranges at our institution as follows:

Hemoglobin: 11.6–15 g/dL

Platelet counts: 150–400 K/mcL

Eosinophils, absolute: 0-0.8 K/mcL

Alkaline phosphatase: 45–129 U/L

Aspartate transaminase: 10–37 U/L

Alanine transaminase: 5–37 U/L

Interleukin 5: <1 pg/mL

Immunoglobulin E: <214 kU/L

### Histopathology

**Fig. 3:**
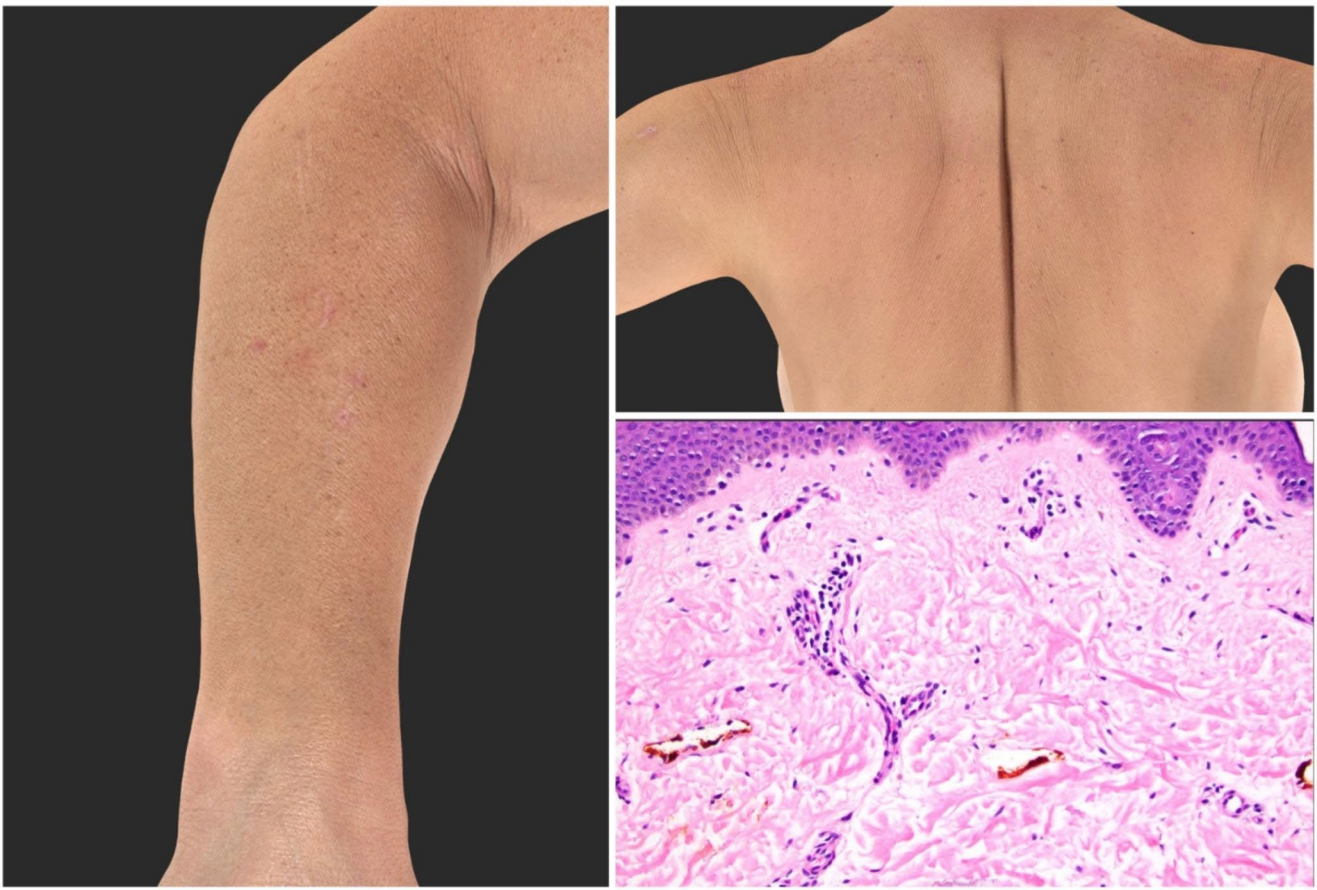
Histopathologic and clinical presentation of excoriations in patients with HP associated pruritus

Four patients received a skin biopsy; three biopsies were available for review. One biopsy showed subtle vacuolar interface changes, one showed a sparse superficial perivascular lymphocytic infiltrate with slight edema and few mast cells (suggestive of an urticarial reaction), and one biopsy showed non-specific features of excoriation with subtle background changes including rare dyskeratotic keratinocytes and slight spongiosis. One biopsy not available for review reportedly showed features of a dermal hypersensitivity reaction. While the biopsy findings are nonspecific, the features are compatible with drug eruptions (Fig. 3).

### Treatment

Fifty-one patients (41.8%) had some treatment for pruritus given by their oncology team (Table 4). Among these patients, antihistamines were the most used (45.1%), followed by topical steroids (39.2%) and emollients (35.3%). Less commonly, systemic steroids (7.8%), gabapentinoids (3.9%), topical anti-itch lotions (3.9%), topical anesthetics (3.9%), and acupuncture (2.0%) were given. The mean number of treatments given by oncology was 1.43 (SD 0.69). Sixty-seven patients (54.9%) were referred to and treated by dermatology. The most common treatments given were topical steroids (71.6%), gabapentinoids (40.3%) and antihistamines (34.3%). Less often, patients received biologics (omalizumab and dupilumab) (13.4%), immunomodulators (11.9%), topical anesthetics (11.9%), topical anti itch lotions (7.5%), ammonium lactate (3.0%), cholestyramine (1.5%), and phototherapy (1.5%). The mean number of treatments given by dermatology was 1.91 (SD 1.25). In total, 101 patients (82.8%) received treatment from oncology and/or dermatology. Of them, thirty-nine (38.6%) were prescribed or directed to use topical medications only. Seventy-four of these patients (60.7%) had descriptions of response to treatment, with 67 (90.5%) experiencing some improvement. The remaining 7 patients (9.5%) did not experience improvement from intervention by oncology or dermatology. Of these, 2 (28.6%) discontinued cancer treatment (1 discontinued their regimen entirely, 1 discontinued pertuzumab only), 2 patients (28.6%) skipped a dose of their regimen until pruritus symptoms abated, and 3 patients (42.8%) experienced improvement in symptoms after completion of their HP regimen as scheduled. Both patients who discontinued HP were switched to a different regimen and were not rechallenged later with HP.

**Table 4:** Treatments for pruritus (n = 122)

		n (%)
Treatments given by oncology	Antihistamine	23 (18.9)
	Topical steroid	20 (16.4)
	Emollients	18 (14.8)
	Systemic steroid	4 (3.3)
	Gabapentinoid	2 (1.6)
	Topical anti-itch	2 (1.6)
	Topical anesthetics	2 (1.6)
	Acupuncture	1 (0.8)
	None	71 (58.2)
Number of oncologist’s treatments for DAEs	0	71 (58.2)
	1	34 (27.9)
	2	13 (10.7)
	3	3 (2.5)
	4	1 (0.8)
Average number of oncology treatments, when treated	Mean (SD)	1.43 (0.69)
Treatments given by dermatology	Topical steroid	48 (39.3)
	Gabapentinoid	27 (22.1)
	Antihistamine	23 (18.9)
	Biologics	9 (7.4)
	Topical anesthetics	8 (6.6)
	Aprepitant	6 (4.9)
	Topical anti-itch	5 (4.1)
	Apremilast	2 (1.6)
	Ammonium lactate	2 (1.6)
	Cholestyramine	1 (0.8)
	Phototherapy	1 (0.8)
	None	55 (45.1)
Number of dermatologic treatments for pruritus	None	55 (45.1)
	1	32 (26.2)
	2	19 (15.6)
	3	10 (8.2)
	4 or more	9 (7.4)
Average number of dermatology treatments, when treated	Mean (SD)	1.91 (1.25)
Treatments reported effective by patients	Topical steroid	35 (28.7)
	Antihistamine	20 (16.4)
	Emollients	14 (11.5)
	Gabapentinoid	11 (9.0)
	Biologics	3 (2.5)
	Systemic steroid	2 (1.6)
	Acupuncture	1 (0.8)
	Topical anti-itch	1 (0.8)
	Ammonium lactate	1 (0.8)
	Topical anesthetics	1 (0.8)
	Phototherapy	1 (0.8)
	No response/response not documented	55 (45.1)

The most effective treatments given by oncology and/or dermatology were also determined from clinical documentation. Thirty-seven patients (50%) experienced improvement with topicals only. Improvement in symptoms was attributed to topical steroids (52.2%), antihistamines (29.9%), emollients (20.9%), gabapentinoids (16.4%), biologics (4.5%), systemic steroids (3.0%), acupuncture (1.5%), topical anti-itch lotions (1.5%), ammonium lactate (1.6), topical anesthetics (1.5%), and phototherapy (1.5%).

## Discussion

In this single center study, we found a reported pruritus incidence of 4.72% among patients treated with HP. This is lower than what has been reported in clinical trials, which have ranged from 11 to 17.6% [8, 11, 12]. The discrepancy may be due to our study design. We were limited to data that could be gathered from the electronic medical record, and therefore our results were likely subject to patient underreporting or provider under-documentation. Despite these deficits, it is possible that our results more accurately reflect the true burden of HP associated pruritus. In clinical trials, patients are systematically assessed with questionnaires and may report mild or unrelated symptoms that would otherwise not be reported in the standard clinical setting. As such, we expect that, although patients with very mild or self-resolving cases may not have been included in our cohort, we adequately characterized patients with symptoms requiring intervention, which is likely more relevant for real world management.

Pruritus among these patients tends to be low grade - only one patient in our cohort experienced a high-grade toxicity. This is consistent with what has been reported in the literature [8, 11, 12]. The most common severity we observed was grade 2, which indicates that despite being low grade, these symptoms may nevertheless lead to limitations in instrumental activities of daily living (ADLs) [13]. Pruritus, even without any associated skin eruption, has been shown to significantly reduce quality of life [14–16]. Fortunately, symptoms among our patients appeared to be manageable with interventions by oncologists and dermatologists, with only 4 (3.45%) requiring treatment interruption or discontinuation. In addition, a large subset of our cohort was managed effectively with topical interventions only. Topical steroids, oral antihistamines, emollients and gabapentinoids appeared to have the most success, which is consistent with management recommendations for pruritus secondary to other targeted therapies [16]. Among our cohort, first generation antihistamines were most often prescribed. Nighttime dosing of these medications was often recommended and may be beneficial given the risk of associated drowsiness.

Unlike cutaneous toxicities associated with other targeted therapies, which typically emerge within the first few cycles or months of therapy, pruritus associated with HP therapy tended to present late [17, 18]. While the mechanism of this is not entirely clear, we speculate that the administration of chemotherapies prior to HP alone may play a role. One hundred and thirteen patients (97.4%) received some cytotoxic chemotherapy, prior to and/or concurrent with HP therapy. The use of these chemotherapies can induce a state of immunosuppression which may impede or delay the development of pruritus or rash. Paclitaxel use was especially common in our cohort, with 93.1% using prior to or concurrent with HP alone. The relationship between paclitaxel and the immune system is a matter of current study, but previous investigators have found that this therapy can inhibit T cell (and therefore autoreactive T cell), B cell or inflammatory cell activation and proliferation, thereby exhibiting autoimmune effects [19, 20].

The mechanism of pruritus development in these patients has not been determined. Absolute eosinophils, IL5 and IgE were within normal range for most of our patients, but it is difficult to draw conclusions from this as circulating levels may not correlate with those in the skin. In considering other etiologies of itch in our cohort, we evaluated for causes of generalized pruritus including uremia, cholestasis, thrombocythemia and iron deficiency anemia. Only a small subset of patients had evidence of impaired renal or liver function, and thrombocytosis was similarly uncommon. Hemoglobin levels were below normal in 39 patients, suggesting the potential role of iron deficiency anemia in the development of itch symptoms among our cohort. Iron tests were not obtained from our patients, but iron deficiency anemia occurs frequently among patients with solid tumors [21, 22]. Histopathologic analysis from our cohort was nonspecific, and, as standard of care, tended to favor patients with concomitant rash. As such, these findings may not be representative of the larger cohort, most of whom did not have any associated eruption.

A notable finding of our study was the anatomic distribution of pruritus, which predominantly affected the upper extremities. In our clinical experience, patients often present with a distribution of itch akin to that seen in brachioradial pruritus, which involves the C5 and C6 dermatomes. Brachioradial pruritus is believed to be due to a combination of cervical nerve irritation and ultraviolet radiation, though its mechanism has yet to be fully elucidated [23]. There is one case in the literature describing a patient with breast cancer (on treatment with HP) who developed brachioradial pruritus. In this particular patient, the development of symptoms was attributed to metastatic disease to her cervical spine [24]. In our cohort, the presence of cervical disease (either metastases or other degenerative pathology) was only documented for 2 patients (1 metastases and 1 degenerative). However, the true number of patients with cervical pathologies or degenerative changes is likely higher than this, especially considering the advanced age of our cohort. Patients in our study were only imaged at our center for evaluation of cancer progression, so degenerative changes may have been underreported or not detected.

In addition to cervical nerve irritation, we can also speculate that there may be some component of photosensitivity in our patients, but there are no existing reports in the literature of photosensitivity due to anti-HER2 therapies specifically. There are, however, reports of photosensitive eruptions secondary to EGFR inhibitors, and both pertuzumab and, to a lesser degree, trastuzumab have been shown to interact with EGFR as heterodimers [25–27]. The disruption of this process attenuates EGFR signaling, and while functional HER2 heterodimers have not been found in human skin, the clinical similarities between anti HER2 and anti EGFR eruptions suggest there may nonetheless be some functional interaction [9]. However, patients in our cohort did not experience pruritus preferentially in the summer months, as may be expected with a photosensitive process. In addition, skin and nail infections secondary to HP therapy appeared histologically similar to EGFR associated cutaneous toxicities, suggesting some shared pathologic process [10]. Further investigation into the origin of this pruritus distribution is needed.

There were some limitations to our study. Many patients did not have a full description of symptoms in their EMR, and therefore we may not have fully captured their histories or treatments. In addition, the descriptive nature of this study limited the analyses that could be performed. We did not have a comparator group, and therefore were unable to comment on patient characteristics or disease features that were associated with the development of pruritus. We were also unable to evaluate the relationship between pruritus development and tumor response to HP. The development of cutaneous toxicities from targeted therapies as a predictor for prognosis and response to treatment has long been a point of interest. This relationship is most well described among patients on immune checkpoint inhibitors, but a similar phenomenon was previously demonstrated among patients receiving anti-HER2 therapies. Using data from the CLEOPATRA study, investigators found that occurrence of pertuzumab rash was associated with improved prognosis for both progression free survival and overall survival [23]. The relationship between pruritus specifically and prognosis was not explored in this study and poses a potential subject for future investigations.

## Data Availability

The datasets are not publicly available but can be provided for researchers who request it from the authors.
